# Lea’s Series of Pocket Text-Books.—Venereal Diseases

**Published:** 1902-02

**Authors:** 


Lea's Series of Pocket Text-Books.—Venereal Diseases.—A manual for students and practitioners. By James R. Haydon, M. D., Chief of Clinic and Instructor in Venereal and Genito-Urinary Diseases at the College of Physicians and Surgeons (Columbia University), New York; Assistant Visiting Genito-Urinary Surgeon to Bellevue Hospital. Third and revised edition. Illustrated with sixty-six engravings. Lea Brothers & Co., Philadelphia and New York. 1901.

The author gives in a clear, concise form an excellent and complete outline of the diagnosis, pathology and treatment of venereal diseases. He wisely omits statistics and matters of purely historical interest. From this little work the student can obtain a practical working knowledge of gonorrhea, stricture, chancroid and syphilis, together with their complications and sequelæ. R.

